# Impact of Long-term Fasting on Breath Volatile Sulphur Compounds, Inflammatory Markers and Saliva Microbiota Composition

**DOI:** 10.3290/j.ohpd.b5795653

**Published:** 2024-10-24

**Authors:** Alexandre Loumé, Franziska Grundler, Françoise Wilhelmi de Toledo, Catherine Giannopoulou, Robin Mesnage

**Affiliations:** a Researcher and Teaching Assistant, University Clinics of Dental Medicine (CUMD), Division of Regenerative Dental Medicine and Periodontology, Geneva, Switzerland. Conceptualisation, investigation, reviewed and edited the manuscript.; b Researcher, Buchinger Wilhelmi Clinic, Überlingen, Germany. Conceptualisation, reviewed and edited the manuscript.; c Senior Scientific Advisor, Buchinger Wilhelmi Clinic, Überlingen, Germany. Conceptualisation, investigation, data curation, reviewed and edited the manuscript, project administration.; d Professor, University Clinics of Dental Medicine (CUMD), Division of Regenerative Dental Medicine and Periodontology, Geneva, Switzerland. Conceptualisation, reviewed and edited the manuscript.; e Scientific Director, Buchinger Wilhelmi Development & Holding, Überlingen, Germany. King’s College London, Department of Nutritional Sciences, London, UK. Conceptualisation, formal analysis, visualisation, wrote, edited and reviewed the manuscript.; * Equal contributions.

**Keywords:** bad breath, gingival crevicular fluid, inflammatory markers, prolonged fasting, saliva microbiota

## Abstract

**Background and Purpose:**

Despite substantial evidence supporting the role of resident bacterial communities in therapeutic fasting outcomes, research has primarily focused on gut microbiota, leaving changes in oral microbiota largely unexplored. The clinical significance of oral health changes during fasting is nonetheless underscored by the documented development of halitosis in fasting individuals. However, no scientific studies have comprehensively examined the interplay between salivary microbiota alterations, inflammatory changes in the gingival crevice, and the production of malodorous volatile compounds. We examined volatile sulphur compounds (VSC) in breath during fasting, cytokine levels in the gingival crevice, and oral microbiota composition of the saliva in a single-arm interventional study involving 36 subjects who fasted for 10 ± 3 days.

**Materials and Methods:**

Participants fasted according to Buchinger fasting guidelines. VSC were evaluated every morning before any food or drink intake using the OralChroma gas chromatography device. Saliva and gingival crevicular fluid (GCF) samples were collected at the clinical site before fasting, at the end of fasting, and at the end of food reintroduction. Follow-up saliva samples were sent to the patients after 1 and 3 months. Saliva samples were processed and analysed by targeted sequencing of 16S rRNA gene amplicons, whereas the expression of 6 inflammatory markers in the GCF were analysed using a multiplex fluorescent bead-based immunoassay.

**Results:**

The quantification of volatile compounds in the breath demonstrated a statistically significant increase in dimethylsulfide levels during fasting, which corroborates the occurrence of bad breath as a common side effect of fasting. Salivary microbiota profiling showed a shift in microbial composition, including reduction in the levels of *Neisseria, Gemella* and *Porphyromonas* spp., concomitant with an increase in the levels of *Megasphaera, Dialister, Prevotella, Veillonella, Bifidobacteria, Leptotrichia, Selenomonas, Alloprevotella*, and *Atopobium*. We further demonstrated a reduction in the levels of the pro-inflammatory cytokine interleukin-8 in the GCF.

**Conclusion:**

Dimethylsulfide concentrations in the breath increased during fasting, and this was correlated to changes in the oral microbiota. Future studies are needed to illuminate the possible impact of these changes on oral and general health status.

Fasting is an effective strategy to control body weight, delay aging and optimise metabolic health. Different forms of fasting have been used in clinical studies, such as intermittent fasting, which lasts less than 2 days, and long-term fasting, which lasts 4 days to several weeks.^42^ Extensive research has demonstrated the systemic effects of long-term fasting on various conditions such as metabolic disorders,^40^ cardiovascular diseases,^16^ hypertension,^15^ oxidative stress^41^ and gut microbiome dysbiosis.^10,29^

Long-term fasting causes mild symptoms (fatigue, sleep problems, dry mouth, back pain, or halitosis), in about 10% of the individuals, mainly in the first days of the fast.^40^ Halitosis, commonly known as bad breath, is frequently reported in individuals engaging in long-term fasting.^40^ These empirical observations made by clinicians have never been scientifically investigated. Changes in breath odour were also reported in published studies in the case of alternate-day fasting,^20^ or during the consumption of a very low-calorie, ketogenic diet.^1^

The production of ketone bodies during fasting can change the smell of fasting people, producing a distinct odour in the mouth. However, the smell of ketones is different from that of pathologic halitosis (i.e., bad breath).^4^ For most patients (80-90%) with bad breath, the cause is found in the mouth.^34^ It is the result of microbial degradation of proteins present in the saliva, food debris and shed epithelial cells. Through this process, a range of volatile compounds are produced of which the volatile sulphur compounds (VSCs) are the most important contributors to bad breath.^24^ Among these, hydrogen sulfide (H_2_S), methylmercaptan (CH_3_SH) and dimethyl sulfide ((CH_3_)_2_SH) represent 90% of the VSCs detected in the breath of patients.

Limited knowledge is available about the effects of fasting on the oral microbiota. After the gut, it is the second largest microbial community in the humans.^2^ Bacteria in the oral cavity colonise both the hard and the soft tissues of teeth and the oral mucosa. These are distinctive niches which provide a unique environment for microbial colonisation. The salivary microbiota has been shown to be individualised and temporally stable in orally healthy individuals.^18,38^ Saliva samples are frequently employed for assessing subgingival microbiota,^12,26^ as numerous clinical studies have documented noteworthy positive correlations between salivary and subgingival microbiota.^3,19,23^

Our group has recently shown that food reintroduction following 10-day periodic fasting, results in statistically significant increases in the levels of four faecal cytokines (IL-6, IL-10, interferon-gamma and TNF-a), suggesting that fasting exerts an immunomodulatory effect.^29^ A clinical study including 47 patients with metabolic syndrome showed that a 2-week fasting protocol reduced bleeding on probing and periodontal inflammation.^32^ In the present study, including a cohort of 36 systemically healthy subjects, our hypothesis is that long-term fasting influences the production of VSC (primary outcome) as a cause of halitosis. In addition, since halitosis is closely linked to oral microbiota dysbiosis and the development of periodontal disease, we explored the oral microbiota composition and the levels of inflammation markers in GCF before and after a 10- ± 3-day fasting period.

## MATERIALS AND METHODS

### Study Design

This prospective, single-arm interventional study was performed at the Buchinger Wilhelmi Clinic (BWC), Überlingen, Germany from September to January 2023. The protocol was approved by the Ethics Committee of the State Medical Association of Baden-Württemberg (F-2022-025 on 1.06.2022) and registered in clinicaltrials.gov (NCT05449249). Written informed consent was obtained from all participants. The study consisted of five main visits. A data and sample collection appointment before and at the end of fasting, as well as at the end of food reintroduction, was conducted at the BWC. Additionally, daily data collections and halitosis measurements were done during the stay. Two follow-up appointments were realized remotely after one and three months, and saliva samples were shipped to BWC.

### Participants

Participants were recruited from the patient cohort of the BWC. Systemically healthy volunteers over 18 years of age, registered for a stay of more than 10 days at the BWC, and completing the multidisciplinary fasting program were included. The presence of a contraindication to fasting, such as cachexia, anorexia nervosa, advanced kidney, liver or cerebrovascular insufficiency, dementia or other severely debilitating cognitive disease and pregnancy or lactation period, comprised the exclusion criteria. We further excluded participants who smoked, had taken antibiotics within the last 8 weeks or had consumed probiotics within the last 4 weeks, or were >80 years of age.

### Fasting Program

The participants fasted in a specialised facility according to the guidelines of the fasting therapy under continuous medical supervision.^39^ The participants received a low-calorie (600 kcal) vegetarian diet of either rice and vegetables or fruits served in three meals one day before the fasting period. The fasting started with the emptying of the intestinal tract through the intake of a laxative (20–40 g NaSO_4_ dissolved in 0.5 L water or Laxoberal; A. Nattermann & Cie., Frankfurt, Germany). During the fasting period, participants received 250 ml organic fresh fruit juice at noon, 250 ml vegetable soup in the evening and 20 g of honey. This resulted in a total daily energy intake of 250 kcal. Water and herbal tea were offered ad libitum and participants were advised to drink at least 2.5 l per day. Every second day, an enema or a mild laxative was applied. The fasting was accompanied by physical activity, alternating with rest, in an environment that promotes calmness and mindfulness. The fasting period was followed by a reintroduction of solid food. The reintroduction occurred stepwise from 800 to 1600 kcal/d and lasted up to 4 days.

### Clinical Parameters

Anthropometric measurements were measured by trained nurses every morning according to the BWC standards. Body weight was assessed with a scale (Seca 704, Seca; Hamburg, Germany) while participants were lightly dressed. Blood pressure and heartrate were measured once on the non-dominant arm in sitting position after a 5-minute rest (boso Carat professional; Bosch+Sohn; Jungingen, Germany). Waist circumference was measured with a measuring tape (Openmindz; Heidelberg, Germany) placed centrally between the lowest rib and the iliac crest before and at the end of fasting. Height was measured with a Seca 285. Blood samples were drawn before and at the end of the fasting period by certificated medical technical assistants in the morning between 7:00 am and 9:30 am. Analysis of the laboratory parameters was conducted at the Medical Center (MVZ) in Ravensburg, Germany, as described in detail previously.^40^ Emotional and physical well-being were evaluated using a visual analogue score (0-10).

### Dental History, Oral Hygiene Standardisation and Periodontal Examination

At baseline and before periodontal examination, information regarding dental history was obtained by questionnaire and included oral hygiene habits, frequency of dental appointments, and previous dental treatments. Each patient’s oral hygiene was standardised. They brushed their teeth with the same brand of toothpaste and toothbrush (the majority of them used dental floss or interdental brushes). No mouthwash was to be used during the study period. Saliva and gingival crevicular fluid collection as well as VSC tests were carried out between 08:00 and 12:00 noon, by the same operator. Participants were instructed to undertake neither any oral hygiene measures nor ingest any snacks at least 1 h prior to the first tests. Similarly, tongue brushing was not allowed to avoid interference with VSC measurements during both the fasting and the food reintroduction phases. After that, a full-mouth clinical examination was assessed at six sites per tooth by means of the following parameters: plaque index score (evaluated at four points, mesial-distal-vestibular-lingual/palatal),^35^ probing pocket depth (PD) and bleeding on probing (BOP), recorded as present within 30 s (1) or absent (0). No radiographic assessment was performed.

### Saliva Microbiota and Analysis

Saliva was collected by spitting twice within 1 min into a sterile tube containing DNA/RNA Shield Reagent. The samples collected before fasting, at the end of fasting, and at the end of the food reintroduction were collected at the clinical site. Follow-up samples were sent by the patients by mail after 1 month (n = 32) and 3 months (n = 28). The samples were stored frozen at -80°C. The samples were processed and analysed with the Microbiome Analysis Service: Targeted Sequencing (Zymo Research Europe; Freiburg, Germany). DNA extraction was performed using a ZymoBIOMICS-96 MagBead DNA Kit (Zymo Research; Irvine, CA, USA). The DNA samples were prepared for targeted sequencing with the Quick-16STM NGS Library Prep Kit (Zymo Research, Irvine, CA). These primers were custom designed by Zymo Research to provide the best coverage of the 16S gene while maintaining high sensitivity. This project used Quick-16STM Primer Sets V1-V2 (Zymo Research Europe). The 16s V1-V2 primer sequences were 27f (AGRGTTYGATYMTGGCTCAG, 20 bp) and 341r (CTGCWGCCHCCCGTAGG, 17 bp). The sequencing library was prepared using an innovative library preparation process in which PCR reactions were performed in real-time PCR machines to control cycles and therefore limit PCR chimera formation. The final PCR products were quantified with qPCR fluorescence readings and pooled together based on equal molarity. The final pooled library was cleaned with the Select-a-Size DNA Clean & Concentrator (Zymo Research), then quantified with TapeStation (Agilent Technologies; Santa Clara, CA, USA) and QuBit (Thermo Fisher Scientific; Waltham, MA). The final library was sequenced on Illumina MiSeq with a v3 reagent kit (600 cycles, 2 x 300 bp). The sequencing was performed with 20% PhiX spike-in.

**Supplementary Fig 1:**
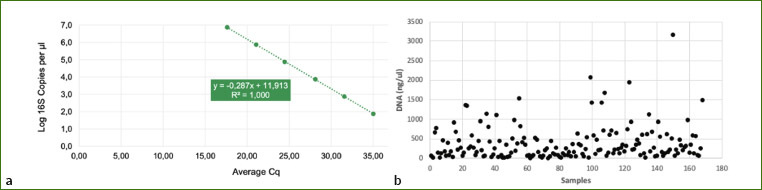
A quantitative real-time PCR was set up with a standard curve. The equation generated by the plasmid DNA standard curve was used to calculate the number of gene copies in the reaction for each sample. The number of genome copies per microliter DNA sample was calculated by dividing the gene copy number by an assumed number of gene copies per genome. The value used for 16S copies per genome is 4. The amount of DNA per microliter DNA sample was calculated using an assumed genome size of 4.64 x 10^6^ bp, the genome size of *Escherichia coli*, for 16S samples. This calculation is Calculated Total DNA = Calculated Total Genome Copies × Assumed Genome Size (4.64 × 106 bp) × Average Molecular Weight of a DNA bp (660 g/mole/bp) ÷ Avogadro’s Number (6.022 x 1023/mole) x DNA Dilution Factor.

To calculate the absolute abundance and estimate biomass, quantitative real-time PCR was conducted using a standard curve, and genome copies per microliter were estimated by dividing the gene copy number by an assumed number of gene copies per genome (Supplementary Fig 1).

### Evaluation of Volatile Sulphur Compounds

VSC were evaluated at each visit in the morning before any food or drink intake using the OralChroma gas chromatography device (OralChroma, Model CHM-2, Insistec; Barcelona, Spain) which makes it possible to determine the levels of the three major VSC associated with halitosis (hydrogen sulfide, methylmercaptan, and dimethyl sulphide). The gas samples were collected with 1-ml disposable syringes which were inserted into each participant’s mouth according to the manufacturer’s instructions.The patients closed their mouths for 60 s, after which 1 ml of air was withdrawn into the syringe. Then, 0.5 ml of air was ejected, and the remaining 0.5 ml of gas was ejected at the entrance of the OralChroma unit. The measurements began automatically. The process required 8 min to complete using the analytical software OralChroma Data Manager.

### Gingival Crevicular Fluid Collection and Analysis

GCF was collected after evaluating the plaque index and before all other periodontal parameters. GCF was collected by means of sterile paper strips (ISO 040, Dentsply Maillefer; Ballaigues, Switzerland) on the mesial-buccal aspect of four teeth, i.e., at one site per quadrant. If available, the 1st molar was chosen. If this tooth was absent, the 2nd molar, 2nd premolar or the 1st premolar was chosen. First, supragingival plaque was carefully removed with cotton pellets and the sites were gently dried with an aspiration tip. After waiting 2 min, the strips were placed at the entrance of the selected sulcus or pocket for 1 min and then transferred to a microtube. The four samples were pooled and stored at -80°C until analysed. Samples contaminated with blood were discarded.

On the day of analysis, each GCF pooled sample was eluted in 80 µl PBS, vortexed for 1 min and then centrifuged for 10 min at 13,000 rpm. Biomarkers in GCF were assessed using a multiplex fluorescent bead-based immunoassay and the Bio-Plex 200 suspension array system (Bio-Rad Laboratories; Hercules, CA, USA). The assays were performed in 96-well filter plates following the manufacturer’s instructions. Six inflammatory markers were measured: interleukin (IL)-1β, IL-6, IL-8, IL-10, interferon-γ (IFN-γ) and tumor necrosis factor-α (TNF-α). The detection limit of the assay varied between 1.0 and 2.2 pg/ml, except for TNF-α (6.6 pg/ml). A constant (0.1) was added to all readings to remove zero values.

### Statistical Analysis

The use of simplified gas chromatography has been shown to be sufficiently sensitive to reflect changes in VSC in lifestyle interventions.^13,22^ We calculated that a sample size of 28 is required to achieve a medium effect-size estimate xxCohen’s d of 0.5 with 80% power and an alpha error probability of 0.05 with a Wilcoxon signed-rank test for matched pairs (G*Power version 3.1.9.7; Heinrich Heine Universty, Düsseldorf, Germany). We thus considered that 36 patients would provide us with sufficient power to detect fasting-related changes in VSC and account for possible missing values (p = 0.05).

Data were analysed using R version 4.0.0 (Vienna, Austria). Concentrations of hydrogen sulfide, methylmercaptan, and dimethyl sulphide were modelled with linear mixed models using the subject unique ID as a random effect and the timepoint as a fixed effect using R package lmerTest. The microbial composition was estimated by calculating the abundance of amplicon sequence variants (ASV) among a collection of sequenced 16S rRNA gene amplicons. The DADA2 pipeline was used to quantify amplicon sequence variants using R.^5^ Pseudo-pooling of samples was performed to increase the sensitivity of the analysis. The xxxtaxonomy was assigned using the native implementation of the naive Bayesian classifier method from DADA2 with the SiLVA ribosomal RNA gene database for the 16S rRNA gene amplicon reads. Cleaned read counts, ASV taxonomiesxx taxa?, and the metadata were then combined for an analysis with the phyloseq package.^28^ The alpha diversity index, estimated as the Shannon index, was used to measure the diversity of the total number of ASVs within the samples. The changes in this alpha diversity index were modelled using linear mixed models as described above. The beta diversity was estimated from the microbial abundance. This was used to calculate Bray-Curtis dissimilarity distances which were analysed with a PERMANOVA test (permutation analysis of variance) to compare the microbial diversity at different timepoints. Linear mixed models were fitted to analyse the differences in microbiota composition between time points for each taxon and of the associations between taxa abundance and health parameters with MaAsLin2 (R package).^25^ The Benjamini and Hochberg correction procedure was used to control the false discovery rate.

## RESULTS

**Fig 1:**
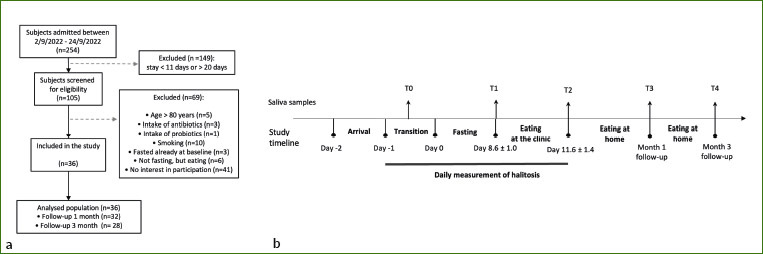
Study design. Flow chart of the selection criteria (a) and flowchart of sample collection (b).

**Supplementary Fig 2:**
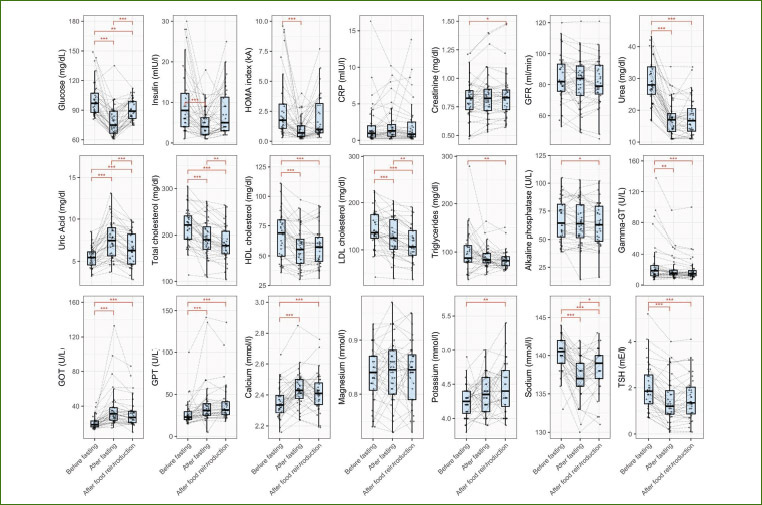
Serum biochemistry parameters measured during long-term fasting confirmed the energy metabolism switch from exogenous glucose to endogenously derived substrates.

**Supplementary Fig 3:**
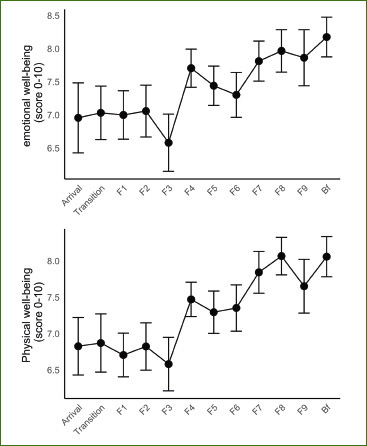
Emotional and physical well-being increased during fasting (visual analogue scores).

We screened 254 subjects for eligibility and subsequently excluded 218 of them. Thirty-six subjects participated in the study (Fig 1, Table 1). Gingivitis was present in only one participant and periodontitis in 40% (Table 1). The remaining 57% had a healthy periodontium defined as absence of sites with PD > 3 mm and BOP > 10%. No adverse effects were recorded, except for a 69-year-old man who had acute ischuria which appeared on the first day of food reintroduction. He was treated with a bladder catheter for three days and tamsulosin. The adverse event was not considered to be related to the fasting intervention and the man continued his participation in the study. The metabolism of participants switched from the use of food-derived substances to endogenously derived substrates, as reflected by a marked a decrease in blood sugar (p = 5.9e-14), insulin (p = 6.8e-5), cholesterol (p = 3.4e-10), gamma-GT (p = 0.001), and uric acid (p = 0) levels (Supplementary Fig 2). Emotional and physical well-being increased during fasting (p<0.05) (Supplementary Fig 3).

**Table 1:** Baseline characteristics of the cohort and self-reported oral health habits and dental history

	
Females, n (%)	26 (72%)
Age, years	61.4 ± 11.2
Fasting length, days [min-max]	8.6 [6–10]
Food reintroduction length, days [min-max]	3 [1–4]
Weight, kg (mean ± SD)	75.2 ± 21.6
Waist circumference, cm (mean ± SD)	90.8 ± 16.7
Systolic blood pressure, mmHg (mean ± SD)	125.4 ± 16.3
Diastolic blood pressure, mmHg (mean ± SD)	83.3 ± 13.1
Toothbrushing, N (%)	
Never	1 (3%)
Once a day	4 (11%)
Twice a day	19 (53%)
>2 times a day	12 (33%)
Approximal tooth cleaning, N (%)	
Never	13 (36%)
Once a week	15 (42%)
Once a day	8 (22%)
Frequency of dental recalls, N (%)	
Never	1 (3%)
Once a year	8 (22%)
Twice a year	15 (42%)
>2 times a year	12 (33%)
Periodontal parameters	Value
Number of teeth, mean ± SD	26.6 ± 1.68
Number of sites Pl = 1, mean ± SD	24.2 ± 18.35
Number of sites Pl = 2, mean ± SD	24.1 ± 23.5
Number of sites Pl = 3, mean ± SD	7 ± 15
Number of sites PD ≥ 4mm	11.37 ± 15.5
Number of sites BOP+, mean ± SD	20.9 ± 23.2
Number of sites PD ≥ 4 + BOP	4.47 ± 9.76
Diagnosis of periodontal disease	
Healthy periodontium	20 (57%)
Gingivitis	1 (2.8%)
Periodontitis	14 (40%)
Mean ± SD: mean value with standard deviation; min-max: range of values.	

**Fig 2:**
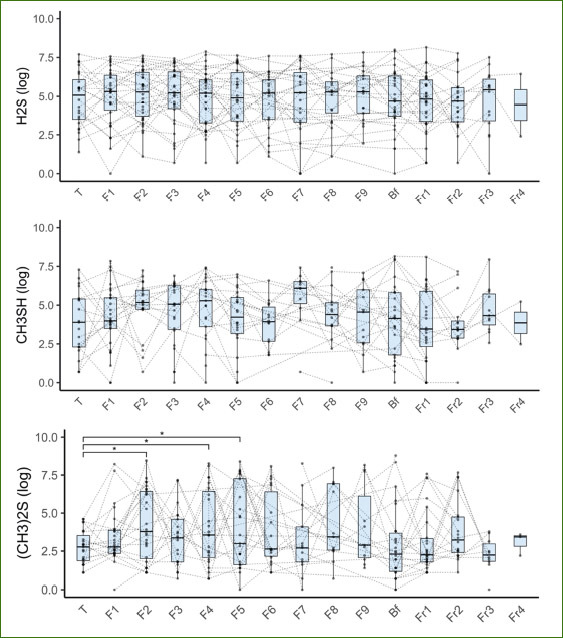
Changes in the levels of volatile sulphur compounds in oral breath during long-term fasting. VSC were measured daily during the transition (T), fasting days (Fx), the day when patients broke their fast (Bf) and the food reintroduction days. Too few patients fasted more than 10 days for their data to be included in the statistical model (n = 36, * p < 0.05).

The quantification of VSCs in the breath during long-term fasting revealed a statistically significant increase in dimethylsulfide ((CH_3_)_2_S) levels. Concentrations in dimethylsulfide increased by 10% and 30% from the baseline to the first and the second day of fasting, respectively. In contrast, no changes were observed in concentrations of hydrogen sulfide (H_2_S) and methyl mercaptan (CH_3_SH) (Fig 2 and Supplementary Table 1).

**Supplementary Table 1:** Changes in the levels of volatile sulphur compounds in oral breath during long-term fasting. VSC were measured daily during the transition (T), fasting days (Fx), the day when patients broke their fast (Bf) and the food reintroduction days

Day	H_2_S	CH_3_SH	(CH_3_)_2_S
T	5.29 ± 1.61	3.72 ± 2	2.9 ± 1.01
F1	5.44 ± 1.52	3.84 ± 1.68	3.19 ± 1.52
F2	6 ± 1.57	4.43 ± 1.84	3.79 ± 1.74
F3	5.35 ± 2	4.46 ± 1.87	3.49 ± 1.08
F4	5.72 ± 1.52	4.64 ± 1.85	2.99 ± 1.61
F5	5.66 ± 1.6	4.13 ± 1.86	3.81 ± 2.71
F6	4.83 ± 1.86	3.55 ± 1.52	2.62 ± 1.16
F7	5.64 ± 2.86	4.59 ± 2.11	3.35 ± 1.27
F8	5.71 ± 1.61	4.58 ± 1.48	3.91 ± 1.91
F9	5.98 ± 1.26	4.07 ± 1.84	3.45 ± 2.06
Bf	5.26 ± 1.81	3.75 ± 2.33	2.7 ± 1.7
Fr1	5.28 ± 1.43	3.41 ± 1.95	2.85 ± 1.67
Fr2	5.26 ± 1.54	3.86 ± 1.86	2.72 ± 0.88
Fr3	5.48 ± 1.62	4.38 ± 1.3	2.34 ± 1.24
Fr4	4.42 ± 2.86	3.86 ± 1.94	3.51 ± 0.11

The concentrations (ppb) are provided as mean ± SD.

**Fig 3:**
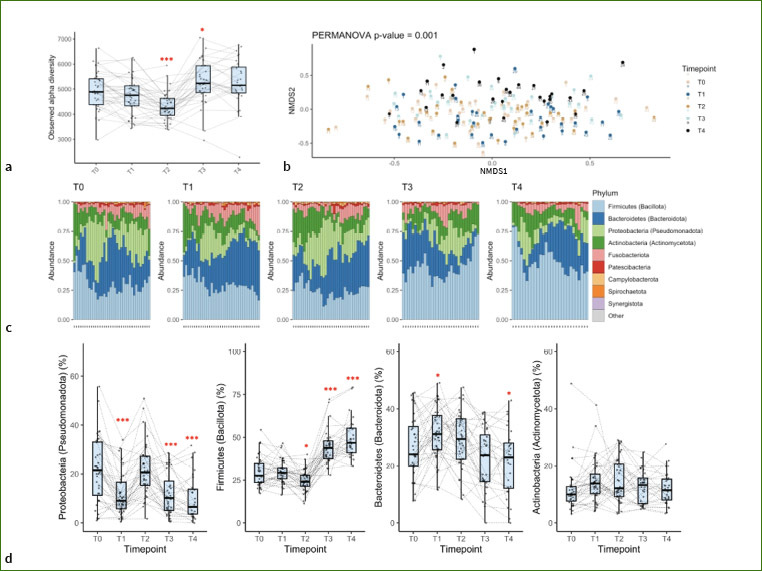
Changes in the saliva microbiota during long-term fasting, after a food reintroduction, and 1 or 3 months later. a. alpha diversity is measured as the total number of observed ASVs (T0: baseline; T1: end of fasting; T2: end of food reintroduction; T3: 1 month later; T4: 3 months later). b. Non-metric Multidimensional Scaling (NMDS) of Bray-Curtis distances shows inter-individual variations in salivary microbiota composition. c. Bar plots representing the total relative abundance. d. Statistical analysis of the changes in saliva microbiota composition (n = 36, *p < 0.05; **p < 0.01; ***p < 0.001).

**Supplementary Fig 4:**
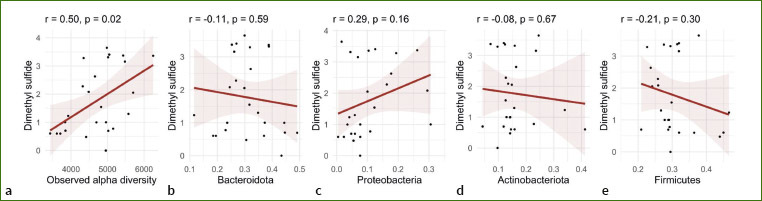
Correlations between the levels of dimethyl sulfide in the breath with the levels of different bacteria at the phylum level in the saliva.

The changes in the saliva microbiota were assessed. Alpha diversity was decreased by fasting (Fig 3a) but was then higher than at baseline during the follow-ups at 1 and 3 months. The dimethyl sulfide levels measured in the participants’ breath correlated with alpha diversity, suggesting that overall bacterial diversity could contribute to the production of this gas (Supplementary Fig 4). PERMANOVA of beta diversity (p = 0.001) revealed that the salivary microbiota changed composition during the study (Fig 3b). Fasting decreased the levels of proteobacteria, and this decrease was still observed after 1 and 3 months, although the proteobacteria levels returned to baseline levels after the food reintroduction (Figs 3c and 3d). Interestingly, levels of Firmicutes (Bacillota) increased substantially during the follow-up examinations, becoming the dominant taxonomic group, while Bacteroidetes (Bacteroidota) and Proteobacteria (Pseudomonadota) were suppressed. Actinobacteria (Actinomycetota) were not affected, despite representing a substantial proportion of the salivary microbiota.

**Fig 4:**
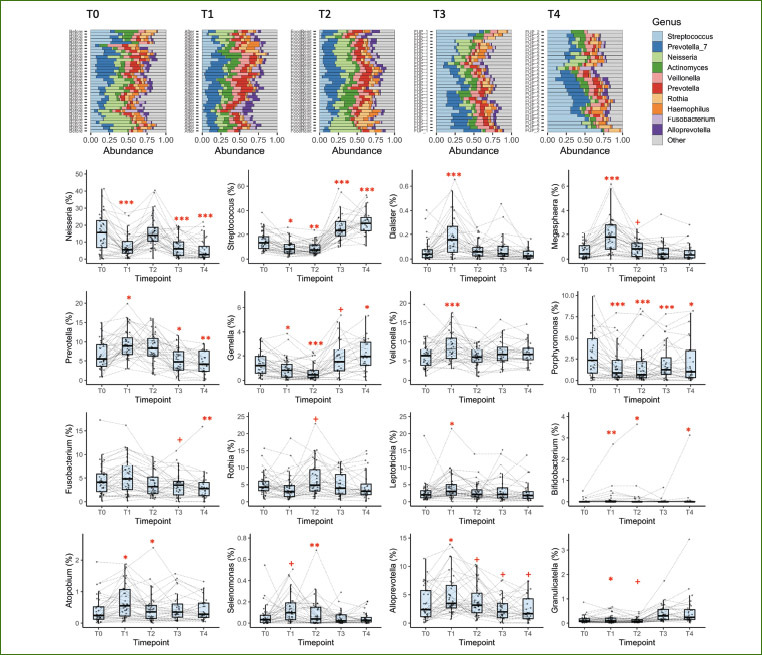
Changes in the saliva microbiota during long-term fasting, after food reintroduction, and 1 and 3 months later. Box plots representing the total relative abundance at the genus level (top panels) (T0: baseline; T1: end of fasting; T2: end of food reintroduction; T3: 1 month later; T4: 3 months later). Statistical analysis of the changes in saliva microbiota composition (n = 36, +, < 0.10; * p < 0.05; **p < 0.01; ***p < 0.001).

We further investigated genus taxonomic compositions (Fig 4). Not every bacterium had its taxonomyxx assigned to the ASV level, thus we focused on genus level (Supplementary Table 2). The changes in Proteobacteria composition during the fast were mostly driven by a decrease in the abundance of the *Neisseria*. There was also a decrease in the abundance of *Gemella* spp. and *Porphyromonas* spp. Interestingly, the reintroduction of the food did not affect these bacteria in a similar fashion. Although the amount of *Gemella* spp. increased in comparison to baseline, the levels of *Porphyromonas* spp. remained lower than at baseline. The sequence variants from *Porphyromonas* spp. were assigned to *Porphyromonas pasteri*. Most of the taxa which were found to become more abundant through the fasting intervention were in the genera *Megasphaera, Dialister, Prevotella, Veillonella, Bifidobacteria, Leptotrichia, Selenomonas, Alloprevotella*, and *Atopobium*. The increase in Firmicutes (Bacillota) abundance observed during the follow-up was mostly attributed to increase in the abundance of various *Streptococcus* and *Gemella* spp.

**Supplementary Table 2:** Statistically significant differences in comparison to the baseline for the comparison of taxonomic abundance at the genus level

Genus	Timepoint	Coefficient ± Std Err	qval
*Abiotrophia*	After	-0.0079 ± 0.0044	2.3E-01
*Abiotrophia*	FoodReint	-0.0133 ± 0.0043	1.8E-02
*Abiotrophia*	FUP_1	0.0119 ± 0.0044	4.6E-02
*Abiotrophia*	FUP_2	0.0138 ± 0.0046	2.4E-02
*Actinobacillus*	After	-0.0396 ± 0.0062	1.9E-07
*Actinobacillus*	FoodReint	-0.029 ± 0.006	1.2E-04
*Actinobacillus*	FUP_1	-0.0158 ± 0.0061	6.3E-02
*Actinobacillus*	FUP_2	-0.0204 ± 0.0064	1.5E-02
*Actinomyces*	After	0.0719 ± 0.0185	2.5E-03
*Actinomyces*	FoodReint	0.0286 ± 0.018	3.2E-01
*Actinomyces*	FUP_1	0.0285 ± 0.0184	3.3E-01
*Actinomyces*	FUP_2	0.0208 ± 0.0192	5.2E-01
*Aggregatibacter*	After	-0.008 ± 0.0045	2.4E-01
*Aggregatibacter*	FoodReint	-0.0083 ± 0.0044	2.1E-01
*Aggregatibacter*	FUP_1	-0.0074 ± 0.0045	3.0E-01
*Aggregatibacter*	FUP_2	-0.008 ± 0.0047	2.8E-01
*Alloprevotella*	After	0.0257 ± 0.0127	1.7E-01
*Alloprevotella*	FoodReint	0.0036 ± 0.0124	8.8E-01
*Alloprevotella*	FUP_1	-0.0367 ± 0.0126	3.0E-02
*Alloprevotella*	FUP_2	-0.0433 ± 0.0132	1.2E-02
*Alloscardovia*	After	0.0139 ± 0.0028	8.0E-05
*Alloscardovia*	FoodReint	0.0076 ± 0.0027	3.9E-02
*Alloscardovia*	FUP_1	0.0007 ± 0.0028	9.0E-01
*Alloscardovia*	FUP_2	0.0034 ± 0.0029	4.8E-01
*Alysiella*	After	-0.0021 ± 0.0018	4.7E-01
*Alysiella*	FoodReint	0.0016 ± 0.0017	5.8E-01
*Alysiella*	FUP_1	-0.0032 ± 0.0018	2.3E-01
*Alysiella*	FUP_2	-0.0029 ± 0.0019	3.3E-01
*Amnipila*	After	-0.0016 ± 0.0014	4.7E-01
*Amnipila*	FoodReint	0.0016 ± 0.0013	4.8E-01
*Amnipila*	FUP_1	0 ± 0.0014	9.9E-01
*Amnipila*	FUP_2	-0.002 ± 0.0014	4.0E-01
*Anaeroglobus*	After	0.0056 ± 0.0016	7.7E-03
*Anaeroglobus*	FoodReint	0 ± 0.0016	9.9E-01
*Anaeroglobus*	FUP_1	0.0022 ± 0.0016	4.0E-01
*Anaeroglobus*	FUP_2	0.0002 ± 0.0017	9.5E-01
*Atopobium*	After	0.0179 ± 0.0042	7.7E-04
*Atopobium*	FoodReint	0.0024 ± 0.0041	7.4E-01
*Atopobium*	FUP_1	0.0084 ± 0.0042	1.8E-01
*Atopobium*	FUP_2	0.005 ± 0.0044	4.8E-01
*Bergeyella*	After	-0.0162 ± 0.0048	1.0E-02
*Bergeyella*	FoodReint	-0.0063 ± 0.0047	4.1E-01
*Bergeyella*	FUP_1	0.0014 ± 0.0048	8.8E-01
*Bergeyella*	FUP_2	-0.0039 ± 0.005	6.3E-01
*Bifidobacterium*	After	0.0134 ± 0.0037	4.9E-03
*Bifidobacterium*	FoodReint	0.0102 ± 0.0036	3.4E-02
*Bifidobacterium*	FUP_1	0.0055 ± 0.0036	3.5E-01
*Bifidobacterium*	FUP_2	0.0115 ± 0.0038	2.3E-02
*Bulleidia*	After	0.0015 ± 0.001	3.7E-01
*Bulleidia*	FoodReint	0.0004 ± 0.001	8.4E-01
*Bulleidia*	FUP_1	0.0018 ± 0.001	2.2E-01
*Bulleidia*	FUP_2	0.0022 ± 0.001	1.6E-01
*Butyrivibrio*	After	0.011 ± 0.0031	5.4E-03
*Butyrivibrio*	FoodReint	0.0159 ± 0.003	1.8E-05
*Butyrivibrio*	FUP_1	-0.0017 ± 0.003	7.6E-01
*Butyrivibrio*	FUP_2	-0.003 ± 0.0032	5.8E-01
*Campylobacter*	After	0.0067 ± 0.0056	4.7E-01
*Campylobacter*	FoodReint	0.0209 ± 0.0054	2.8E-03
*Campylobacter*	FUP_1	-0.0106 ± 0.0055	2.0E-01
*Campylobacter*	FUP_2	-0.0116 ± 0.0058	1.8E-01
*Candidatus.Saccharimonas*	After	-0.0031 ± 0.0031	5.6E-01
*Candidatus.Saccharimonas*	FoodReint	-0.0068 ± 0.0031	1.2E-01
*Candidatus.Saccharimonas*	FUP_1	-0.0089 ± 0.0031	3.3E-02
*Candidatus.Saccharimonas*	FUP_2	-0.0088 ± 0.0033	4.9E-02
*Capnocytophaga*	After	0.0137 ± 0.008	2.8E-01
*Capnocytophaga*	FoodReint	0.0256 ± 0.0078	1.2E-02
*Capnocytophaga*	FUP_1	-0.002 ± 0.008	9.0E-01
*Capnocytophaga*	FUP_2	-0.0097 ± 0.0083	4.8E-01
*Cardiobacterium*	After	0.0094 ± 0.0027	7.5E-03
*Cardiobacterium*	FoodReint	0.0116 ± 0.0026	5.2E-04
*Cardiobacterium*	FUP_1	-0.0008 ± 0.0027	8.7E-01
*Cardiobacterium*	FUP_2	-0.0022 ± 0.0028	6.3E-01
*Catonella*	After	0.0086 ± 0.0054	3.3E-01
*Catonella*	FoodReint	0.0119 ± 0.0053	1.2E-01
*Catonella*	FUP_1	0.0021 ± 0.0054	8.3E-01
*Catonella*	FUP_2	-0.0056 ± 0.0057	5.6E-01
*Centipeda*	After	0.0357 ± 0.0076	1.7E-04
*Centipeda*	FoodReint	0.0251 ± 0.0074	9.2E-03
*Centipeda*	FUP_1	-0.0064 ± 0.0075	6.1E-01
*Centipeda*	FUP_2	-0.0065 ± 0.0079	6.2E-01
*Conchiformibius*	After	-0.0015 ± 0.0019	6.3E-01
*Conchiformibius*	FoodReint	0.0017 ± 0.0019	5.9E-01
*Conchiformibius*	FUP_1	0 ± 0.0019	9.9E-01
*Conchiformibius*	FUP_2	0.0023 ± 0.002	4.9E-01
*Corynebacterium*	After	0.007 ± 0.0058	4.7E-01
*Corynebacterium*	FoodReint	0.0121 ± 0.0057	1.4E-01
*Corynebacterium*	FUP_1	0.0071 ± 0.0058	4.7E-01
*Corynebacterium*	FUP_2	0.0056 ± 0.0061	5.8E-01
*Defluviitaleaceae.UCG.011*	After	-0.001 ± 0.0011	5.8E-01
*Defluviitaleaceae.UCG.011*	FoodReint	0.0005 ± 0.0011	8.1E-01
*Defluviitaleaceae.UCG.011*	FUP_1	-0.0015 ± 0.0011	4.0E-01
*Defluviitaleaceae.UCG.011*	FUP_2	-0.0012 ± 0.0011	5.4E-01
*Dialister*	After	0.0183 ± 0.0028	1.2E-07
*Dialister*	FoodReint	0.0035 ± 0.0027	4.4E-01
*Dialister*	FUP_1	0.0042 ± 0.0028	3.4E-01
*Dialister*	FUP_2	-0.0024 ± 0.0029	6.2E-01
*Eikenella*	After	0.0124 ± 0.0023	1.6E-05
*Eikenella*	FoodReint	0.0034 ± 0.0022	3.5E-01
*Eikenella*	FUP_1	-0.0004 ± 0.0023	9.1E-01
*Eikenella*	FUP_2	-0.0011 ± 0.0024	8.0E-01
*Erysipelotrichaceae.UCG.007*	After	-0.0046 ± 0.0028	3.0E-01
*Erysipelotrichaceae.UCG.007*	FoodReint	-0.0024 ± 0.0027	6.0E-01
*Erysipelotrichaceae.UCG.007*	FUP_1	0.0004 ± 0.0027	9.3E-01
*Erysipelotrichaceae.UCG.007*	FUP_2	0.0005 ± 0.0029	9.2E-01
F0058	After	0.0108 ± 0.0028	3.1E-03
F0058	FoodReint	0.0053 ± 0.0028	2.0E-01
F0058	FUP_1	-0.002 ± 0.0028	6.7E-01
F0058	FUP_2	-0.0031 ± 0.003	5.4E-01
F0332	After	-0.0015 ± 0.0028	7.6E-01
F0332	FoodReint	-0.0035 ± 0.0027	4.3E-01
F0332	FUP_1	0.002 ± 0.0027	6.6E-01
F0332	FUP_2	0.0061 ± 0.0029	1.4E-01
Family.XIII.UCG.001	After	0.0044 ± 0.0017	5.8E-02
Family.XIII.UCG.001	FoodReint	0.0021 ± 0.0016	4.4E-01
Family.XIII.UCG.001	FUP_1	-0.0002 ± 0.0017	9.5E-01
Family.XIII.UCG.001	FUP_2	-0.0016 ± 0.0018	5.8E-01
Filifactor	After	0.0031 ± 0.0026	4.8E-01
*Filifactor*	FoodReint	-0.0012 ± 0.0026	8.0E-01
*Filifactor*	FUP_1	-0.0031 ± 0.0026	4.7E-01
*Filifactor*	FUP_2	-0.0027 ± 0.0027	5.6E-01
*Fretibacterium*	After	0.0067 ± 0.0029	1.0E-01
*Fretibacterium*	FoodReint	0.0032 ± 0.0028	4.9E-01
*Fretibacterium*	FUP_1	-0.0041 ± 0.0028	3.8E-01
*Fretibacterium*	FUP_2	-0.0031 ± 0.003	5.4E-01
*Fusobacterium*	After	0.0141 ± 0.0109	4.4E-01
*Fusobacterium*	FoodReint	-0.0194 ± 0.0106	2.3E-01
*Fusobacterium*	FUP_1	-0.0263 ± 0.0108	8.3E-02
*Fusobacterium*	FUP_2	-0.0394 ± 0.0114	7.5E-03
*Gemella*	After	-0.0242 ± 0.0082	2.7E-02
*Gemella*	FoodReint	-0.0382 ± 0.008	1.3E-04
*Gemella*	FUP_1	0.0187 ± 0.0081	1.0E-01
*Gemella*	FUP_2	0.0281 ± 0.0085	1.1E-02
*Granulicatella*	After	-0.0018 ± 0.0039	8.0E-01
*Granulicatella*	FoodReint	-0.0059 ± 0.0038	3.2E-01
*Granulicatella*	FUP_1	0.0221 ± 0.0038	3.1E-06
*Granulicatella*	FUP_2	0.0244 ± 0.004	8.1E-07
*Haemophilus*	After	-0.0552 ± 0.0133	1.2E-03
*Haemophilus*	FoodReint	-0.0144 ± 0.0129	5.0E-01
*Haemophilus*	FUP_1	-0.0357 ± 0.0132	4.8E-02
*Haemophilus*	FUP_2	-0.0425 ± 0.0138	2.0E-02
*Johnsonella*	After	0.0125 ± 0.0038	1.1E-02
*Johnsonella*	FoodReint	0.0038 ± 0.0037	5.4E-01
*Johnsonella*	FUP_1	0.0004 ± 0.0037	9.5E-01
*Johnsonella*	FUP_2	-0.0006 ± 0.0039	9.3E-01
*Kingella*	After	0.007 ± 0.0036	2.0E-01
*Kingella*	FoodReint	0.009 ± 0.0036	7.0E-02
*Kingella*	FUP_1	-0.0032 ± 0.0036	6.0E-01
*Kingella*	FUP_2	-0.0049 ± 0.0038	4.4E-01
*Lachnoanaerobaculum*	After	0.0149 ± 0.0063	9.6E-02
*Lachnoanaerobaculum*	FoodReint	0.015 ± 0.0062	8.3E-02
*Lachnoanaerobaculum*	FUP_1	0.0054 ± 0.0063	6.1E-01
*Lachnoanaerobaculum*	FUP_2	0.0006 ± 0.0066	9.5E-01
*Lautropia*	After	-0.0074 ± 0.0082	5.9E-01
*Lautropia*	FoodReint	0.0073 ± 0.008	5.9E-01
*Lautropia*	FUP_1	-0.0038 ± 0.0082	8.0E-01
*Lautropia*	FUP_2	-0.0095 ± 0.0085	5.0E-01
*Lentimicrobium*	After	0.0023 ± 0.0014	3.0E-01
*Lentimicrobium*	FoodReint	-0.0006 ± 0.0013	8.0E-01
*Lentimicrobium*	FUP_1	-0.0017 ± 0.0014	4.4E-01
*Lentimicrobium*	FUP_2	-0.0015 ± 0.0014	5.4E-01
*Leptotrichia*	After	0.0444 ± 0.0145	2.1E-02
*Leptotrichia*	FoodReint	0.0077 ± 0.0141	7.6E-01
*Leptotrichia*	FUP_1	0.0143 ± 0.0144	5.6E-01
*Leptotrichia*	FUP_2	-0.0003 ± 0.0151	9.9E-01
*Megasphaera*	After	0.0581 ± 0.0061	2.8E-14
*Megasphaera*	FoodReint	0.0146 ± 0.0059	7.8E-02
*Megasphaera*	FUP_1	-0.0034 ± 0.006	7.6E-01
*Megasphaera*	FUP_2	-0.0054 ± 0.0063	6.1E-01
*Mobiluncus*	After	0.0072 ± 0.002	7.0E-03
*Mobiluncus*	FoodReint	0.0107 ± 0.002	1.6E-05
*Mobiluncus*	FUP_1	0.0008 ± 0.002	8.4E-01
*Mobiluncus*	FUP_2	0.0005 ± 0.0021	9.1E-01
*Mycoplasma*	After	0.0027 ± 0.0017	3.2E-01
*Mycoplasma*	FoodReint	-0.0017 ± 0.0016	5.3E-01
*Mycoplasma*	FUP_1	0.0007 ± 0.0017	8.2E-01
*Mycoplasma*	FUP_2	-0.0006 ± 0.0017	8.5E-01
*Neisseria*	After	-0.1235 ± 0.0253	9.9E-05
*Neisseria*	FoodReint	0.0204 ± 0.0247	6.2E-01
*Neisseria*	FUP_1	-0.1336 ± 0.0251	1.8E-05
*Neisseria*	FUP_2	-0.1809 ± 0.0263	2.9E-08
*Olsenella*	After	0.0025 ± 0.0017	3.7E-01
*Olsenella*	FoodReint	0.0007 ± 0.0017	8.2E-01
*Olsenella*	FUP_1	0.0006 ± 0.0017	8.7E-01
*Olsenella*	FUP_2	0.0004 ± 0.0018	9.1E-01
*Oribacterium*	After	0.0075 ± 0.0079	5.7E-01
*Oribacterium*	FoodReint	0.0035 ± 0.0077	8.0E-01
*Oribacterium*	FUP_1	0.0018 ± 0.0079	9.0E-01
*Oribacterium*	FUP_2	-0.0073 ± 0.0082	6.0E-01
*Ottowia*	After	0.0119 ± 0.0034	6.5E-03
*Ottowia*	FoodReint	0.0131 ± 0.0033	2.0E-03
*Ottowia*	FUP_1	-0.001 ± 0.0034	8.8E-01
*Ottowia*	FUP_2	-0.0018 ± 0.0035	7.8E-01
*Parvimonas*	After	0.0114 ± 0.004	3.3E-02
*Parvimonas*	FoodReint	-0.0046 ± 0.0039	4.7E-01
*Parvimonas*	FUP_1	0.0047 ± 0.004	4.8E-01
*Parvimonas*	FUP_2	0.0019 ± 0.0041	8.0E-01
*Peptococcus*	After	0.0059 ± 0.0026	1.2E-01
*Peptococcus*	FoodReint	-0.0027 ± 0.0026	5.4E-01
*Peptococcus*	FUP_1	-0.0021 ± 0.0026	6.3E-01
*Peptococcus*	FUP_2	-0.0038 ± 0.0027	4.0E-01
*Peptostreptococcus*	After	0.01 ± 0.0062	3.1E-01
*Peptostreptococcus*	FoodReint	-0.0012 ± 0.006	9.1E-01
*Peptostreptococcus*	FUP_1	0.0016 ± 0.0061	8.9E-01
*Peptostreptococcus*	FUP_2	-0.0022 ± 0.0064	8.5E-01
*Porphyromonas*	After	-0.0513 ± 0.0112	2.6E-04
*Porphyromonas*	FoodReint	-0.0479 ± 0.0109	5.1E-04
*Porphyromonas*	FUP_1	-0.036 ± 0.0111	1.3E-02
*Porphyromonas*	FUP_2	-0.0435 ± 0.0116	3.6E-03
*Prevotella*	After	0.0427 ± 0.0142	2.4E-02
*Prevotella*	FoodReint	0.0258 ± 0.0139	2.2E-01
*Prevotella*	FUP_1	-0.041 ± 0.0141	3.0E-02
*Prevotella*	FUP_2	-0.0593 ± 0.0148	1.7E-03
*Prevotella_7*	After	0.0081 ± 0.0188	8.1E-01
*Prevotella_7*	FoodReint	0.0044 ± 0.0183	9.0E-01
*Prevotella_7*	FUP_1	-0.0226 ± 0.0186	4.7E-01
*Prevotella_7*	FUP_2	-0.0323 ± 0.0195	3.0E-01
*Pseudopropionibacterium*	After	-0.0027 ± 0.0028	5.6E-01
*Pseudopropionibacterium*	FoodReint	0.0017 ± 0.0027	7.2E-01
*Pseudopropionibacterium*	FUP_1	0.0033 ± 0.0027	4.7E-01
*Pseudopropionibacterium*	FUP_2	0.0012 ± 0.0029	8.2E-01
Rikenellaceae.RC9.gut.group	After	0.0014 ± 0.0017	6.1E-01
Rikenellaceae.RC9.gut.group	FoodReint	-0.0017 ± 0.0016	5.3E-01
Rikenellaceae.RC9.gut.group	FUP_1	-0.0021 ± 0.0016	4.3E-01
Rikenellaceae.RC9.gut.group	FUP_2	-0.0009 ± 0.0017	7.6E-01
*Rothia*	After	-0.0165 ± 0.0158	5.4E-01
*Rothia*	FoodReint	0.0373 ± 0.0154	8.3E-02
*Rothia*	FUP_1	-0.0017 ± 0.0157	9.5E-01
*Rothia*	FUP_2	-0.0098 ± 0.0164	7.4E-01
*Selenomonas*	After	0.0168 ± 0.0038	4.3E-04
*Selenomonas*	FoodReint	0.0068 ± 0.0037	2.2E-01
*Selenomonas*	FUP_1	0.0007 ± 0.0037	9.1E-01
*Selenomonas*	FUP_2	0.0011 ± 0.0039	8.8E-01
*Solobacterium*	After	0.002 ± 0.0033	7.4E-01
*Solobacterium*	FoodReint	-0.0019 ± 0.0032	7.5E-01
*Solobacterium*	FUP_1	0.0054 ± 0.0033	3.1E-01
*Solobacterium*	FUP_2	-0.0004 ± 0.0035	9.5E-01
*Stomatobaculum*	After	0.0015 ± 0.0076	9.1E-01
*Stomatobaculum*	FoodReint	-0.0054 ± 0.0075	6.7E-01
*Stomatobaculum*	FUP_1	0.0119 ± 0.0076	3.3E-01
*Stomatobaculum*	FUP_2	0.0099 ± 0.008	4.5E-01
*Streptococcus*	After	-0.0643 ± 0.0205	1.8E-02
*Streptococcus*	FoodReint	-0.0749 ± 0.02	3.6E-03
*Streptococcus*	FUP_1	0.1519 ± 0.0204	2.1E-09
*Streptococcus*	FUP_2	0.219 ± 0.0214	9.2E-16
*Tannerella*	After	0.0083 ± 0.0034	8.1E-02
*Tannerella*	FoodReint	-0.0026 ± 0.0033	6.4E-01
*Tannerella*	FUP_1	-0.0083 ± 0.0034	8.1E-02
*Tannerella*	FUP_2	-0.0116 ± 0.0036	1.2E-02
TM7a	After	0.0006 ± 0.0021	8.8E-01
TM7a	FoodReint	-0.0018 ± 0.002	6.0E-01
TM7a	FUP_1	0.0006 ± 0.0021	8.9E-01
TM7a	FUP_2	0.0019 ± 0.0022	6.1E-01
TM7x	After	0.0076 ± 0.0069	5.0E-01
TM7x	FoodReint	0.0056 ± 0.0067	6.1E-01
TM7x	FUP_1	-0.0041 ± 0.0068	7.4E-01
TM7x	FUP_2	-0.0094 ± 0.0071	4.3E-01
*Treponema*	After	0.0146 ± 0.0046	1.5E-02
*Treponema*	FoodReint	0.006 ± 0.0045	4.1E-01
*Treponema*	FUP_1	-0.0025 ± 0.0045	7.6E-01
*Treponema*	FUP_2	-0.007 ± 0.0048	3.7E-01
*Veillonella*	After	0.0422 ± 0.0104	1.6E-03
*Veillonella*	FoodReint	-0.0049 ± 0.0102	8.0E-01
*Veillonella*	FUP_1	0.0175 ± 0.0104	2.9E-01
*Veillonella*	FUP_2	0.015 ± 0.0108	4.0E-01
X.Eubacterium..brachy.group	After	0.0053 ± 0.0023	9.6E-02
X.Eubacterium..brachy.group	FoodReint	0.0008 ± 0.0022	8.4E-01
X.Eubacterium..brachy.group	FUP_1	0.0062 ± 0.0023	4.6E-02
X.Eubacterium..brachy.group	FUP_2	0.007 ± 0.0024	2.7E-02
X.Eubacterium..nodatum.group	After	-0.005 ± 0.0049	5.4E-01
X.Eubacterium..nodatum.group	FoodReint	-0.0037 ± 0.0047	6.3E-01
X.Eubacterium..nodatum.group	FUP_1	-0.0006 ± 0.0048	9.4E-01
X.Eubacterium..nodatum.group	FUP_2	-0.0074 ± 0.005	3.7E-01
X.Eubacterium..saphenum.group	After	0.0007 ± 0.0014	8.0E-01
X.Eubacterium..saphenum.group	FoodReint	-0.0002 ± 0.0014	9.2E-01
X.Eubacterium..saphenum.group	FUP_1	-0.001 ± 0.0014	6.6E-01
X.Eubacterium..saphenum.group	FUP_2	0.0008 ± 0.0015	7.8E-01
X.Eubacterium..yurii.group	After	0.0086 ± 0.0021	1.7E-03
X.Eubacterium..yurii.group	FoodReint	0.0044 ± 0.0021	1.5E-01
X.Eubacterium..yurii.group	FUP_1	-0.0015 ± 0.0021	6.7E-01
X.Eubacterium..yurii.group	FUP_2	-0.0014 ± 0.0022	7.3E-01

Bacterial genera which are considered to be statistically significant with q-values (qval) below 5% are indicated in bold characters, and whether their levels is decreased (red) or increased (green) in comparison to baseline (coefficient ± SD) is indicated by a colour coding.

**Fig 5:**
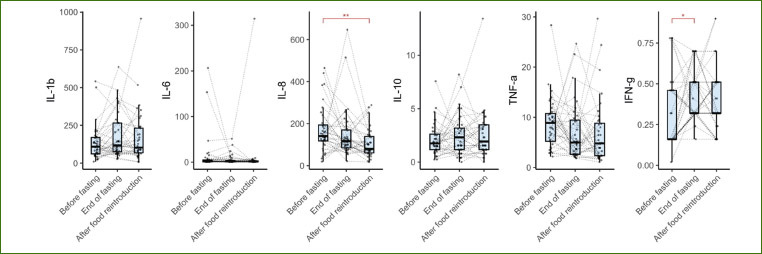
Gingival crevicular fluid cytokine levels over the course of a long-term fasting intervention. Variations in cytokine levels were measured before fasting, at the end of fasting, and at the end of the food reintroduction (n = 36, *p < 0.05; **p < 0.01; ***p < 0.001).

The assessment of oral inflammation markers in the GCF demonstrated a statistically significant reduction in the levels of interleukin-8 following prolonged fasting (p = 0.11 at the end of fasting, p = 0.009 at the end of the food reintroduction). In contrast, there was a marginal increase in the levels IFN-γ (p = 0.03) at the end of fasting as compared to baseline (Fig 5).

The raw data of the oral microbiota analysis will be posted on the NCBI Sequence Read Archive upon publication. All the other raw data will be made available upon request.

## DISCUSSION

In the present study of 36 systemically healthy individuals, dimethylsulfide concentrations in the breath increased during fasting. This increase in DMS was found to be correlated with changes in bacterial diversity in the oral microbiota, suggesting that alterations in oral microbiota composition during long-term fasting might contribute to the development of halitosis. However, it is important to note that our study does not definitively confirm DMS as the sole gas responsible for these changes, nor does it establish the oral cavity as the exclusive source of its production. Hydrogen sulfide, methyl mercaptan and, to a lesser extent, dimethylsulfide, account for 90% of the VSC in bad breath.^24^ Studies have shown the presence of bacterial species that are known to produce DMS in the human gastrointestinal tract^30^ and in the oral cavity.^31^ The increase in DMS and associated bad breath symptoms may be temporary and linked to the metabolism of sulphur-containing aminoacids during fasting.^27^ DMS is stable in blood and can be transported into breath.^37^ The generation of odorous compounds is an intricate process, and attributing halitosis solely to a single microbial species is not feasible.

Fasting may impact oral health by temporarily eliminating food intake. The absence of food can provide a restorative break for the oral cavity. This cessation of chewing, mechanical lesions, and the absence of nutrients in saliva might offer the gingiva a chance to recover, similar to the digestive rest that promotes intestinal regeneration during fasting.^10^ Fasting also caused substantial body weight loss, which was shown to impact the composition of the salivary microbiota with comparable variations in bacterial compositions, such as a decrease in *Gemella* and *Porphyromonas* and an increase in *Veillonella* and *Megasphaera.*^11^

An important factor that may contribute to the positive outcomes of therapeutic fasting is the subsequent shift towards a healthier diet. Our prior research has indicated that individuals tend to adopt more health-conscious dietary choices, such as opting for organic foods^17^ and increasing their consumption of fiber-rich food.^29^ Wright et al^43^ demonstrated that individuals who adhered to a dietary pattern rich in fruits, vegetables, salads, water, and tea, while limiting their consumption of fermentable carbohydrates, fatty acids, protein, and sugary beverages, exhibited a lower prevalence of periodontal disease. Furthermore, a randomised controlled trial even demonstrated that supplementing the diet with concentrated fruit, vegetable, and berry juices during standard non-surgical periodontal therapy resulted in improved reductions in pocket depth.^7^

The decrease in the levels of *Porphyromonas* spp. suggests a reduction in the levels of potential pathogens by fasting.^36^ However, it must be borne in mind that drawing definitive conclusions about the health implications of changes in salivary bacteria composition during extended fasting requires a deeper examination at the species or even strain level. For instance, although *Streptococcus oralis* ssp. *dentisani* is known to be a core member of the salivary microbiota contributing to oral health,^33^ some *Streptococcus oralis* strains are pathogens contributing to periodontal diseases and can even cause bactaeremia and endocarditis.^8^ The virulence and pathogenicity of bacteria, especially within the diverse *Streptococcus mitis* group, can often be attributed to single genes.^44^

The effects of fasting on the saliva microbiota were less pronounced compared to known effects on the gut microbiota.^10^ This is likely to be due to the resilience of oral microbial communities to external perturbations. Stability is a key feature of the human oral microbiome.^18,38^ It is known to be less susceptible to major changes or disruptions by external environmental factors at the individual level. When two individuals were sampled over the course of an entire year, 95% of the operational taxonomic units of the oral bacterial population were found to be stable over the course of the study, while only the minor components of the microbiota were found to be involved in fluctuations.^9^ This was in complete contrast to the gut microbiota, which was statistically significantly perturbed by dietary influences, antibiotic usage, and other lifestyle factors. In another study comparing 22 different body sites in 236 healthy adults as part of the Human Microbiome Project, the oral microbiota was found to be the most temporally stable microbial community in the body.^45^ This stability, at least in health, may be a function of the dominant and continuous influence of saliva in the nutrition of oral bacteria, the impact of diet being minimal – except in the case of overwhelming quantities of readily metabolised fermentable dietary sugars.^45^

The potential implications of our study for the clinical practice of therapeutic fasting in addressing periodontal health remain a subject of debate. Another finding in the present study was the decrease in the levels of IL-8 in the GCF following therapeutic fasting. IL-8 is a pro-inflammatory cytokine that plays a crucial role in the recruitment and activation of neutrophils, contributing to the inflammatory response in periodontal diseases.^14^ A recent study demonstrated that the efficacy of periodontal treatment is directly linked to a drop in salivary IL-8 levels.^21^ The observed reduction in IL-8 levels in our study suggests that therapeutic fasting may have anti-inflammatory effects in the oral cavity.

It is important to acknowledge the main limitations of our study, such as the relatively small sample size, the absence of a matched group that did not fast (control), and the lack of diagnosis of periodontal disease based on the current classification of periodontal disease and conditions.^6^ Furthermore, the direct impact of fasting on the periodontal tissues, in terms of periodontal inflammation, was not evaluated. To better understand the clinical implications of therapeutic fasting, further research involving larger and longer-term longitudinal studies is needed. Including participants with diverse dietary backgrounds and health statuses could also help assess how individual differences influence the observed effects of fasting on oral health. Including more timepoints could also help decipher the cascade of events, for instance, whether the increase in volatile compounds would precede the shifts observed in microbiota, in turn preceding the decrease observed in IL-8. These future investigations can help validate our findings and shed light on the potential long-term effects of fasting on periodontal health.

## CONCLUSION

Our comprehensive investigation into the effects of long-term fasting on oral health underscores the importance of considering dietary interventions like fasting in the context of oral disease management. It opens avenues for further investigation into the mechanistic underpinnings of fasting-related oral health improvements and its potential applications in clinical practice. As the field of fasting research continues to evolve, our findings contribute to the growing body of knowledge aimed at enhancing not only overall health but also the well-being of the oral cavity.
